# Vaccinations for Elite Athletes

**DOI:** 10.3390/vaccines13090931

**Published:** 2025-08-31

**Authors:** Olli Ruuskanen, Maarit Valtonen, Olli J. Heinonen, Matti Waris, Jussi Mertsola

**Affiliations:** 1 Department of Paediatrics and Adolescent Medicine, Turku University Hospital and University of Turku, PL 52, 20521 Turku, Finland; olli.ruuskanen@tyks.fi; 2 Finnish Institute of High Performance Sport KIHU, 40700 Jyväskylä, Finland; 3 Paavo Nurmi Centre and Unit for Health and Physical Activity, University of Turku, 20520 Turku, Finland; olli.heinonen@utu.fi; 4 Institute of Biomedicine, University of Turku, 20520 Turku, Finland

**Keywords:** vaccination, athlete, sport, COVID-19, influenza, pertussis, RSV vaccine, pneumococcal vaccine

## Abstract

Elite athletes are at an increased risk of infections due to behavioral and social factors and frequent travel. Furthermore, heavy physical exercise may induce immunosuppression. Most infections in athletes are acute respiratory illnesses (ARIs) with various viral etiologies. Although athletes, as young, healthy adults, are not at risk for severe infections, a prolonged ARI may ruin a training season or a significant competition or may spread within a sports team. Many common infections are vaccine-preventable. This Opinion advocates for more active vaccination among athletes, although some of the vaccines are not officially recommended for young adults. New respiratory syncytial virus (RSV) protein vaccines are effective and well-tolerated. Yearly influenza and COVID-19 vaccinations are strongly recommended. Conjugated polyvalent pneumococcal vaccines are recommended because they may also induce protection against respiratory viral infections. Pertussis and measles outbreaks are occurring globally. The history of measles vaccination should be reviewed, and consideration should be given to a pertussis booster vaccination (Tdap). A recombinant vaccine can effectively prevent herpes zoster. The vaccination of elite athletes is a cost-effective and powerful tool, but it is currently underused. The sports medicine community can address vaccine hesitancy among athletes by listening to their concerns and giving accurate information.

## 1. Introduction

Elite athletes are a specific target for vaccination [1]. Athletes are young, healthy adults and are not at risk for severe infections. However, elite athletes play sports professionally, and even a single possibly vaccine-preventable infection may ruin the season or spread rapidly within a sports team, inducing significant economic losses. Elite athletes are vulnerable to contracting infections [2,3]. They train 400–800 h annually, and heavy physical exercise may induce relative immunosuppression [4]. During the COVID-19 pandemic, it was discovered that the risk of infection is most closely associated with contact with the infected individual. Consequently, the most critical risk factors for contracting a viral infection are a closed environment, crowded conditions, and prolonged close contact settings [5,6]. All these risk factors are common in the life of elite athletes, especially during frequent travelling, training camps, and competitions. In one study, it was found that the athletes participating in a major winter sports competition for 14 days had a 7-fold increase in the risk of viral acute respiratory illness (ARI) compared to normally exercising control subjects [7]. In another recent 12-month prospective study, a higher incidence of ARI was detected in elite skiers compared to the controls [8].

The rapid development of SARS-CoV-2 mRNA vaccines, the introduction of new respiratory syncytial virus (RSV) protein vaccines, and recent outbreaks of pertussis and measles have reemphasized the importance of vaccinations for athletes as well. A marked number of athletes are not adequately vaccinated, and vaccination is not taken seriously enough in the sports medicine community [9,10,11,12,13,14]. There are official recommendations of vaccinations for young adults, but we are not aware of any international recommendations on the vaccination for athletes (Table 1) [15]. In this Opinion, primarily addressed to the sports medicine community, we briefly discuss vaccines and vaccine-preventable infections that concern elite athletes.

## 2. Vaccinations for Athletes Not Officially Recommended for Young Healthy Adults

### 2.1. RSV Vaccination

After seven decades of a long and rocky road of vaccine development, two protein subunit vaccines, RSVpreF3OA (Arexvy, GSK, London, England) and RSVpreF (Abrysvo, Pfizer, New York, NY, USA), were approved in 2023, and an mRNA vaccine (mResvia, Moderna, Cambridge, MA, USA) was approved in 2024. The primary target group for RSV vaccines is adults aged 60 years and older. Abrysvo is also licensed for the immunization of all individuals over 18 years of age. The efficacy of the vaccines is over 80% and the efficacy lasts above 50% for at least 3 years [16,17,18]. Importantly, in breakthrough infections, RSV vaccination may attenuate the illness [19]. The US Advisory Committee on Immunization Practices recommends the RSV vaccine now for all adults aged at least 75 years and adults aged at least 60 years with a risk of severe infection [15]. In addition, Abrysvo has been approved for use in pregnant women to protect the newborn after birth, when the child is most prone to a severe RSV infection [20]. It is evident that in the future, RSV vaccines will be made available to all individuals. Currently, RSV vaccination outside of the official recommendations should be considered for all elite athletes. Pain and fatigue are the most reported adverse reactions, resolving within 2–3 days [17].

Originally, RSV was found to be a common causative agent of severe lower respiratory tract infections in young children. Nearly all children have suffered RSV infection by the age of 4 years. With the development of sensitive PCR diagnostics, it was found that RSV is also a common cause of lower respiratory tract infections in hospitalized older adults. Today, with the general use of sensitive molecular diagnostics, it is clear that RSV is a common cause of the common cold, i.e., sore throat, rhinitis, and cough, in all age groups. RSV was estimated to cause 5.2 million ARIs in 2019. It induces regular and predictable 3–4-month epidemics in most countries annually during the winter months [21]. Although poorly studied, it is evident that RSV infections are also common in elite athletes. During the 2018 Winter Olympic Games, we identified an RSV cluster in the Finnish team [22]. A probable RSV outbreak in young healthy adults was reported in a military training camp, including 588 febrile illnesses. The main symptoms were sore throat, cough, sputum, and rhinorrhea. The mean duration of fever was 2.3 days [23]. RSV infections have been reported in young adults in several household transmission studies [24,25]. For early detection and prevention of the spread, over-the-counter combined commercial antigen tests are available for the virological diagnosis of RSV, SARS-CoV-2, and influenza (RSV/SARS-CoV-2/Influenza A/B Combo Rapid Antigen Test Kit, HA TECH, Sydney, Australia). There is no antiviral therapy for RSV infection.

### 2.2. COVID-19 Vaccination

The efficacy and safety of multiple SARS-CoV-2 mRNA vaccines were demonstrated in one year, which is a record time. Vaccination was crucial in controlling the pandemic. In an analysis of 68 studies, the effectiveness of a booster vaccine (mostly against Omicron) at baseline (14–42 days) was 70% against infections and reduced to 43% at 112 days or later [26]. The 2024–2025 vaccines were recommended for all persons aged ≥6 months, and at least 8 weeks after the last dose [27]. However, in most countries, yearly COVID-19 booster vaccination is now recommended only for adults over the age of 65 years and for all persons above the age of 6 months with one or more risk factors [28]. The optimal timing of yearly COVID-19 booster vaccination within the Northern Hemisphere is suggested to be from autumn to early winter and can be given together with the influenza vaccine [29]. Uptake of the annual booster has been markedly decreasing [28].

COVID-19 vaccinations are essential for all elite athletes [30]. Due to waning immunity, booster vaccination for elite athletes before major competitions (e.g., the Olympics, World Championships) should be considered. Soon after the vaccine became available, side effects and their impact on sporting participation were reported. In a primary vaccination series study of 127 athletes, the most common side effects were pain around the injection and fatigue. The side effects were more common after the second vaccination, including fever or chills (18%), muscle aches (33%), and headache (34%). Importantly, the side effects lasted 1–2 days and had a minimal impact on the athletes’ ability to train [31]. Myocarditis is a rare adverse event following immunization with COVID-19 mRNA vaccines. Initially, the highest risk was in males aged 12–30 years after the second dose of the primary series. Recently, to evaluate the risk of myocarditis following a booster vaccination in 12- to 39-year-olds, 8.9 million residents were followed for 12,271,861 person-years. The number of cases of myocarditis detected was 1533. On average, 0.9–2.0 males will experience myocarditis hospitalization per 100,000 booster vaccinations [32].

In most cases, myocarditis has been mild and self-limiting [33]. A study including 7911 cases of myocarditis in a period where 130 million vaccine doses were given suggests that a minimum of a 6-month interval after the last dose minimizes the risk of vaccination-associated myocarditis [34]. It is of note that compared to myocarditis associated with COVID-19 illness, vaccination-associated myocarditis occurs less frequently and is less severe [33,35].

Recently, 22 outbreaks of COVID-19 were reported during sports and exercise, suggesting high transmissibility among athletes [36]. The outbreaks were mostly reported in fitness classes and by soccer clubs. The clinical manifestations of COVID-19 in athletes are typically mild, including rhinorrhea, sore throat, fatigue, and headache. The need for hospitalization is infrequent. However, it is possible that the unavailability of athletes for training or competition may last 7–10 days [37,38]. Myocarditis may develop in 1–2% of athletes and necessitates a 3–6-month break from effective training [39,40]. The risk of long COVID (symptoms lasting longer than 3 months) in athletes is small but exists [41].

### 2.3. Pneumococcal Vaccination

There are several different pneumococcal vaccines on the market. Recently, the US Advisory Committee on Immunization Practices (ACIP) has updated its recommendations for the use of pneumococcal polyvalent conjugate vaccines (PCV) among adults. If the vaccination history is unknown or the subject has not received a PCV, a single dose of PCV 21 (Capvaxive, Merck, Rathaway, NJ, USA) or PCV 20 (Prevnar 20, Pfizer, New York, NY, USA) is recommended starting at the age of 50 years and for those aged 19–64 years with certain risk conditions, e.g., cigarette smoking, malignancy, diabetes mellitus, and immunodeficiencies. An additional vaccination is not needed. The commonly solicited adverse reactions include injection site pain, fatigue, headache, and myalgia [15,42].

There is increasing evidence of biological and clinical interaction between pneumococcus and respiratory viruses. The interaction occurs in both directions; viruses affect pneumococci, and pneumococci, in turn, affect viruses. Approximately 10–20% of adults are pneumococcal carriers (in contrast to 90% of young children) [43]. RSV and rhinovirus infections predispose pneumococcal carriage by multiple host-dependent and host-independent mechanisms [44,45]. On the other hand, pneumococcal carriers are more susceptible to acquiring respiratory viruses (including SARS-CoV-2) and, at the time of viral infection, may have an impairment of virus-specific immune responses [45,46,47]. Importantly, pneumococcal conjugate vaccines decrease pneumococcal carriage.

In a seminal study conducted on 37,107 children in South Africa 20 years ago, PCV9 (against nine pneumococcal serotypes) prevented 31% of pneumonias associated with respiratory viruses [48]. A review of 13 studies in children reported PCV efficacy ranging from 41% to 86% against influenza. Three studies in adults reported PCV13 efficacy of 32–35% against SARS-CoV-2, 24–51% against human seasonal coronaviruses, and 13–36% against influenza A [46]. An analysis of 13,856 patients with virus-associated lower respiratory tract infection showed that PCV13 conferred moderate protection against virus-associated illness [49]. Furthermore, PCV13 was associated with modest protection against SARS-CoV-2 infection in 484,801 older adults who had received more than 2 COVID-19 vaccines [50].

*Streptococcus pneumoniae* is the leading cause of bacterial respiratory infections. The clinical manifestations of pneumococcal infections include acute otitis media, sinusitis, pneumonia, and, rarely, sepsis or meningitis. All these pneumococcal infections, however, are not common in athletes, i.e., healthy young adults. We recommend pneumococcal vaccination for athletes, especially because, as mentioned, pneumococcal vaccination also has efficacy against respiratory viral infections.

### 2.4. Herpes Zoster Vaccination

A non-live recombinant herpes zoster (shingles) vaccine (glycoprotein E plus adjuvant) (Shingrix, GSK) was licensed in 2017. Its efficacy is over 95%. A two-dose vaccination schedule is recommended for use in immunocompetent adults aged 50 years and older, as well as for those aged 19 years and older who are immunocompromised. The vaccine is well-tolerated, mainly producing mild local reactions. Fever, chills, and fatigue are possible side effects in immunocompromised individuals [51].

The major risk factor for herpes zoster is decreased T-cell immunity, which occurs naturally during aging. It is noteworthy that heavy physical exercise, such as a marathon, temporarily decreases T-cell function and may increase susceptibility to herpes zoster [4,52,53]. We have witnessed herpes zoster in some elite athletes, but the real occurrence in athletes is not known. Herpes zoster vaccination is optional for athletes because they may have a temporary decrease in natural immunity. The major problem with the vaccination is the cost of the vaccine.

Herpes zoster is a painful vesicular rash that most commonly occurs at the lumbar, thoracic, or cervical dermatomes, not crossing the midline. It is caused by the reactivation of the latent varicella-zoster virus. The primary infection has been chickenpox or the live attenuated varicella vaccine. The typical rash is preceded by local pain or itching. The rash lasts 7–10 days. Prolonged local neuralgia may develop in 10–50% of patients and can significantly impact an athlete’s quality of life. Diagnosis of herpes zoster is clinical [54].

## 3. Vaccinations for Athletes Recommended for All Young Adults

### 3.1. Influenza Vaccination

There are three types of influenza vaccines: live attenuated, inactivated whole virus, and subunit vaccines. Current vaccines are multivalent with antigens from influenza A (H1N1), A (H3N2), and B (Victoria lineage) viruses. The influenza vaccine must be given yearly because influenza viruses concurrently change (antigenic drift). It is worth noting that the effectiveness of influenza vaccines against any of the viral subtypes varies yearly, ranging from 10% to 60% [55,56]. In a recent study, the 2022–2023 influenza vaccine effectiveness against laboratory-confirmed influenza illness was 38%, but only 13% against laboratory-confirmed asymptomatic influenza virus infection. Vaccination did not prevent asymptomatic infections, suggesting that influenza vaccination reduces the progression of infection to illness, a finding consistent with COVID-19 vaccinations [57].

Furthermore, influenza vaccination may be associated with illness attenuation [58]. Influenza vaccinations are generally well tolerated, inducing local tenderness at the injection site in most cases. Annual influenza vaccinations are recommended for all athletes. One meta-analysis demonstrated that regular physical activity significantly increased antibody levels after an influenza vaccination [59]. Recently, the US Food and Drug Administration (FDA) approved an at-home nasal spray influenza vaccine, FluMist (AstraZeneca, Cambridge, England), which may increase influenza vaccine use.

Influenza epidemics are predictable. They occur every year in the Northern Hemisphere between December and February and in the Southern Hemisphere between June and August. The annual incidence varies from 3% to 11%, resulting in 3–5 million cases of severe illness and 290,000–650,000 deaths globally [60]. The symptoms of influenza (caused by influenza A or B viruses) typically start abruptly and include a sore throat, a dry cough, and nasal discharge. Up to half of the patients are febrile and may have chills, myalgia, and headache. The symptoms last typically 3–7 days. Influenza may induce non-respiratory complications, e.g., myocarditis and pericarditis [55]. Point-of-care influenza testing (antigen or PCR) is recommended because influenza can be treated or prevented with antivirals [61]. Outbreaks have been reported during major sports events and among sports teams. During the 2002 Winter Olympics in Salt Lake, UT, USA, 36 (19%) of 188 patients who were tested had influenza. A temperature greater than 37.8 °C was recorded in 39% of the patients [62]. During the 2018 Winter Olympic Games in PyeongChang, South Korea, nine different viral infections were detected in the Finnish team (N = 112), including five cases of influenza B and one case of influenza A [22]. Influenza outbreaks occurred in two professional ice hockey teams just after they played two games against each other. Influenza was transmitted to 15–20% of the players, and as a result, they missed important games. A marked number of players were not vaccinated against influenza [63].

### 3.2. Pertussis Vaccination

Acellular pertussis vaccines provide good protection against disease, but this protection is transient, lasting only 4–5 years [64]. That is why regular boosters are recommended. Currently, pertussis booster vaccines are given in conjunction with tetanus and diphtheria vaccines (Tdap) every 10 years [15]. The vaccine is safe, but local pain and swelling may occur occasionally [65].

Pertussis (whooping cough) is an infection of the lungs caused by *Bordetella pertussis* bacteria. Pertussis outbreaks have increasingly occurred globally [66]. Several infants have died from pertussis. It is essential to emphasize that half of the thousands of reported cases have been in adolescents and adults. Consequently, pertussis poses a significant risk of prolonged respiratory symptoms for athletes.

Pertussis is a highly contagious illness. Over 40 years ago, we demonstrated in a family study that 83% of household contacts became infected [67]. The incubation period of pertussis after contracting it is 7–10 days. The clinical manifestations at the onset of illness are those of a common cold: sore throat, runny nose, and cough. A persistent cough develops in a week and may last 2–3 months, and the infection has been called a “100-day cough”. In young adults, a prolonged cough may be the only symptom. The cough is worse at night. Coughing may be so intense that it induces rib fractures and subconjunctival hemorrhages. Young infants are most vulnerable, and a characteristic whooping may be heard after a cough attack during inspiration. PCR and serology are used in diagnostics. Patients should stay at home 48 h after starting antibiotics [65,66].

### 3.3. Measles Vaccination

Live attenuated measles vaccines are available as the sole measles vaccine or associated with rubella and mumps vaccines. Two doses of the measles vaccine in children have an efficacy of 90–95%. It is worth noting that vaccinations are effective even after exposure to measles [68]. In young athletes, especially those with an unknown vaccination history, a booster dose is recommended. The adverse effects of measles vaccination include local pain and fever in 5–15% of the vaccinees [69]. In all athletes, the measles vaccination history should be checked, and if unknown, serologic tests for immunity should be used.

Measles (caused by measles morbillivirus) is an example of a highly contagious and potentially fatal illness that is fully vaccine-preventable. Measles cases, however, are increasing globally (from 8.6 million in 2022 to 10.3 million in 2023) due to a gap in vaccine coverage. In some countries, large outbreaks have been reported [69,70].

The typical clinical manifestations of measles include maculopapular rash, fever, cough, coryza, and conjunctivitis. Measles is contagious 4 days before the onset of symptoms, which may persist up to 3 weeks. Measles is the most infectious virus, and one case of measles can cause 12–18 secondary cases in susceptible populations. Diagnosis of measles is based on IgM serology or detection of viral RNA by PCR [69].

## 4. Vaccinations for Athletes Needed Locally

### 4.1. Meningococcal Vaccination

Meningococcal diseases (caused by the bacterium *Neisseria meningitidis*) are potentially life-threatening infections. The clinical manifestations are mostly meningitis and septicemia. Meningococcal diseases are exceptionally prevalent in sub-Saharan Africa. Local athletes and athletes visiting the area are recommended to be vaccinated (e.g., Penbraya, Pfizer, New York, NY, USA) against the infection (Table 1).

### 4.2. Tick-Borne Encephalitis Vaccination

Tick-borne encephalitis is an infection of the central nervous system caused by the tick-borne encephalitis virus (TBEV). Twenty-five countries in Europe have TBE-endemic areas, with high numbers reported in Czechia, Germany, Lithuania, Sweden, Austria, and Latvia [71]. Most infections are asymptomatic, but symptomatic infections often require hospitalization. Initial symptoms include fever, headache, vomiting, and weakness. After a few days, symptoms of central nervous system involvement may develop, including confusion, difficulty speaking, and seizures. Permanent neurological dysfunction may remain. The inactivated TBE vaccine (FSME-Immun, Baxter, Deerfield, IL, USA) is effective. It is given as a 3-dose series [72,73]. It is recommended for athletes travelling to a TBE-endemic area and having extensive exposure to ticks during outdoor activities, e.g., orienteering and trail-running

### 4.3. Chikungunya Virus Vaccination

Chikungunya virus (CHIKV) induces annual infections in tropical and subtropical regions. It has been estimated from seroprevalence studies that there are 35 million CHIKV infections yearly, mostly in Southeast Asia, Africa, and the Americas. Notably, CHIKV disease is also returning to Europe [74]. The infection is transmitted by mosquitoes. Acute CHIKV infection is characterized by rash, headache, and fever. About 50% of patients will develop joint pain that can last for months [75]. Recently, two CHIKV vaccines (IXCHIQ, Valvena and VIMKUNYA, Bavaria Nordic, Hellerup, Denmark) were licensed both in the United States and Europe. The vaccines are important new tools to control CHIKV outbreaks [75,76].

## 5. Vaccine Hesitance of Elite Athletes

About 20% of people are estimated to be vaccine-hesitant, being unsure about vaccination [77,78,79,80]. There is some evidence that vaccine hesitance may be more common in elite athletes [11,12,78]. It is understandable that professional athletes carefully consider the potential adverse events and effects of vaccination on their athletic performance. However, very few are strongly opposed to vaccination. The sports medicine community should be professional when addressing concerns about vaccination. Three ways have been suggested. First, listen; do not judge. Second, express precise and reliable information. Third, be honest concerning the possible adverse effects and the efficacy of the vaccine [78]. Sports physicians have a key role in vaccinations, with shared decision-making.

## 6. Conclusions

Elite athletes are at risk of acquiring and transmitting vaccine-preventable infections. However, they are often underimmunized. Outbreaks of vaccine-preventable infections, including respiratory syncytial virus infections, COVID-19, influenza, and measles, have been reported in sports teams. An updated vaccination program specific to athletes should be an essential part of their healthcare. Systematically offered immunizations against vaccine-preventable infections would be in the best interest of the athletes. The adverse events are mild or moderate, most often injection site pain. If needed, vaccinations can be administered during a training program but well before competitions [1,11,59,80]. Vaccine hesitancy within sports communities should be approached with scientific evidence and honesty. Sport physicians, coaches, and family members may serve as “trusted messengers” [81].

## Figures and Tables

**Table 1:** Vaccines for bacterial and viral infections for ages 19 years or older, modified from [15].

Vaccine Target(s)	Routine Vaccination	For Elite Athletes
Chikungunya virus	Locally	Locally
COVID-19	>65 years old; risk groups	Recommended
*Haemophilus influenzae* type b	Given in childhood	As general population
Hepatitis A and hepatitis B	Given in childhood; risk groups	Check history
Human papillomavirus	Given in childhood	As general population
Influenza	Yearly	Recommended
Measles, mumps, and rubella	Given in childhood	Check history
Meningococcal infection	Locally	Locally
Mpox	Risk groups, locally	Risk groups, locally
Pneumococcal infection	Given in childhood; >50 years old	Recommended
Poliovirus	Given in childhood	As general population
Respiratory syncytial virus	>60 years old; pregnant persons	Recommended
Tetanus, diphtheria, and pertussis	Given in childhood	Check history
Tick-borne encephalitis virus	Locally	Locally
Varicella	Given in childhood	Check history
Zoster, recombinant	>50 years old	Recommended

## Data Availability

Data are contained within the article.

## Abbreviations

The following abbreviations are used in this manuscript:

ACIP	Advisory Committee on Immunization Practices
ARI	Acute respiratory illness
CDC	Centers for Disease Control and Prevention
CHIKV	Chikungunya virus
COVID-19	Coronavirus disease 2019
FDA	Food and Drug Administration
PCR	Polymerase chain reaction
PCV	Pneumococcal conjugate vaccine
RSV	Respiratory syncytial virus
TBE	Tick-borne encephalitis
